# Recommendations for Mental Health Chatbot Conversations: An Integrative Review

**DOI:** 10.1111/jan.16762

**Published:** 2025-01-22

**Authors:** Heidi Nieminen, Anna‐Kaisa Vartiainen, Raymond Bond, Emilia Laukkanen, Maurice Mulvenna, Lauri Kuosmanen

**Affiliations:** ^1^ Department of Nursing Science University of Eastern Finland Kuopio Finland; ^2^ Department of Health and Social Management University of Eastern Finland Kuopio Finland; ^3^ School of Computing Ulster University Belfast UK; ^4^ Savonia University of Applied Sciences Kuopio Finland

**Keywords:** chatbots, conversational design, health, mental health

## Abstract

**Aim:**

To identify and synthesise recommendations and guidelines for mental health chatbot conversational design.

**Design:**

Integrative review.

**Methods:**

Suitable publications presenting recommendations or guidelines for mental health conversational design were included. The quality of included publications was assessed using Joanna Briggs Institute Critical Appraisal Tools. Thematic analysis was conducted.

**Data sources:**

Primary searches limited to last 10 years were conducted in PubMed, Scopus, ACM Digital Library and EBSCO databases including APA PsycINFO, CINAHL, APA PsycArticles and MEDLINE in February 2023 and updated in October 2023. A secondary search was conducted in Google Scholar in May 2023.

**Results:**

Of 1684 articles screened, 16 publications were selected. Three overarching themes were developed: (1) explicit knowledge about chatbot design and domain, (2) knowing your audience and (3) creating a safe space to engage. Results highlight that creating pleasant and effective conversations with a mental health chatbot requires careful and professional planning in advance, defining the target group and working together with it to address its needs and preferences. It is essential to emphasise the pleasant user experience and safety from both technical and psychological perspectives.

**Conclusion:**

Recommendations for mental health chatbot conversational design have evolved and become more specific in recent years. Recommendations set high standards for mental health chatbots. To meet that, co‐design, explicit knowledge of the user needs, domain and conversational design is needed.

**Implications for the Profession and/or Patient Care:**

Mental health professionals participating in chatbot development can utilise this review. The results can also inform technical development teams not involving healthcare professionals directly.

**Impact:**

Knowledge of developing mental health chatbot conversations appears scattered. In mental health chatbots, features that enhance the chatbot's ability to meet users' needs and increase safety should be considered. This review is useful for developers of mental health chatbots and other health applications used independently.

**Reporting Method:**

This integrative review was reported according to PRISMA guidelines, as applicable.

**Patient or Public Contribution:**

No patient or public contribution.

**Summary**

- What does this paper contribute to the wider global clinical community?
  ○ This review contributes by increasing knowledge of digital healthcare solutions.
  ○ Synthesised recommendations and tips from recent publications are helpful for healthcare professionals participating in chatbot development processes.
  ○ The results can be utilised by technical development teams in which healthcare professionals are not closely involved.

## 1 Introduction

Mental health issues are a growing health problem globally. Despite the high need for mental health services, the response has been insufficient and inadequate . A World Health Organization (WHO) report indicates that one in eight people around the world, 970 million people worldwide, was living with a mental disorder in 2019, with anxiety and depression being the most common issues (WHO 2022). Among the European Union's population only, more than one‐third experience mental health problems every year (Wittchen et al. 2011). Mental health problems are still being perceived as stigmatising, which may be a barrier to seeking help (Arnaez et al. 2020). Previous research also reports other barriers, including preference to handle the problem alone or with friends or family, embarrassment and structural barriers like costs, time, transportation or scheduling (Ebert et al. 2019). Diversifying and scaling up treatment options is one suggested way of tackling these problems. This includes digital solutions, like chatbots, which can help to deliver guided and unguided self‐help and remote care (WHO 2022).

Chatbots are computer programs that facilitate a conversation in which the human user inputs some text and the chatbot provides a response. Chatbot conversations simulate chatting with another human. The chatbot responds to the user's initiative but the chatbot can also follow up with questions to keep the conversation going. Chatbots are widely used in customer service, information retrieval, education and e‐commerce but also increasingly in promoting health and well‐being (McTear, Callejas, and Griol 2016). They are used across many areas of health for information retrieval, facilitating self‐care (Bates 2019), screening symptoms (Moreira et al. 2023) and delivering interventions (Singh et al. 2023).

Mental health is one of the most common areas for using health chatbots (Milne‐Ives et al. 2020; Wilson and Marasoiu 2022). Chatbots are internationally used in therapy, training and screening, for example, in depressive symptoms, anxiety, posttraumatic stress disorder and autism (Abd‐Alrazaq et al. 2019). Chatbots have also been studied in the context of delivering positive psychology skills (Greer et al. 2019), postpartum mental health promotion (Suharwardy et al. 2023), motivating people with eating disorders to seek treatment and utilise mental health services (Shah et al. 2022), supporting students' well‐being (Sia et al. 2021) as well as alleviating academic stress (De Nieva et al. 2020). According to previous research, mental health chatbots have shown potential in treating depressive symptoms (Lim et al. 2022; Limpanopparat, Gibson, and Harris 2024). Literature reviews from recent years also reveal that depression is one of the mental health issues most often targeted by chatbots (Abd‐Alrazaq et al. 2020; Limpanopparat, Gibson, and Harris 2024; Vaidyam, Linggonegoro, and Torous 2021). Drawing conclusions regarding other mental health challenges, for example, anxiety and stress, is more difficult due to the variety of outcome measures used in studies (Abd‐Alrazaq et al. 2020; Limpanopparat, Gibson, and Harris 2024), differences in reporting results and the heterogeneity of chatbot features (Vaidyam, Linggonegoro, and Torous 2021). Even though there are promising results on chatbots supporting mental health (Abd‐Alrazaq et al. 2020; Lim et al. 2022; Limpanopparat, Gibson, and Harris 2024) and they seem to be mainly positively received by the users (Abd‐Alrazaq et al. 2021; Limpanopparat, Gibson, and Harris 2024), there are still contradictions and uncertainties in the results (Abd‐Alrazaq et al. 2020; Limpanopparat, Gibson, and Harris 2024). Hence, more research on their effectiveness (Abd‐Alrazaq et al. 2020; Vaidyam, Linggonegoro, and Torous 2021), acceptability, suitable target groups and the best ways to use mental health chatbots is needed (Vaidyam, Linggonegoro, and Torous 2021).

It is suggested that chatbots could successfully work as an addition alongside traditional treatment (Vaidyam, Linggonegoro, and Torous 2021). A survey by Sweeney et al. (2021) found that mental healthcare professionals see benefits in using chatbots. These benefits include, for example, helping individuals manage their mental health, improving access and timeliness of care and providing more personalised care, as well as assisting mental health professionals with daily tasks (Sweeney et al. 2021). Increasing mental health service needs and rapid technological advancements are likely to drive the use and research of mental health chatbots in the future (Vaidyam, Linggonegoro, and Torous 2021).

There are several ways to build and classify chatbots. Rule‐based chatbots' responses are hand‐crafted based on how the interaction between user and chatbot is anticipated to proceed (Castle‐Green et al. 2020). Retrieval‐based and generative models using machine learning techniques produce more natural and flexible conversations (Adamopoulou and Moussiades 2020). Despite the rapid development of AI, existing health chatbots still mainly rely on decision trees or predefined rules to select responses (Abd‐Alrazaq et al. 2019; Parmar et al. 2022). This allows the health intervention to be completed within certain frames. It also decreases the likelihood of inappropriate responses (Manyika, Silberg, and Presten 2019). In the context of health, the adoption of generative AI has raised questions about responsibility, as well as ethical risks, and the possibility of inaccurate or even harmful responses due to possible bias in the learning data (Meskó and Topol 2023).

Rule‐based chatbots lead the user through the conversation path towards the completed task (Castle‐Green et al. 2020), and according to Mitchell et al. (2021), an automated chatbot can offer a positive coaching experience (Mitchell et al. 2021). However, from the user's perspective, hand‐crafted interaction can feel restricted (Castle‐Green et al. 2020), shallow, confusing or too short (Abd‐Alrazaq et al. 2021). In conversational interfaces, the user experience is primarily formed from the design and sequencing of utterances (Moore and Arar 2019) and it needs to be considered carefully throughout the design process (Castle‐Green et al. 2020). According to Abd‐Alrazaq et al.'s (2021) review, even though mental health chatbots are perceived as useful and easy to use, conversation quality needs to be improved. It is also important to ensure the quality of information (Abd‐Alrazaq et al. 2021).

Multidisciplinary cooperation is important in developing digital health interventions. For example, in 2019, the Nursing and Artificial Intelligence Leadership (NAIL) Collaborative stated that healthcare professionals' understanding and focus on human relationships and empathy, and knowledge of clinical problems can notably contribute to AI application development (Ronquillo et al. 2021). It is also valuable in mental health chatbot design. Co‐designing with user groups and health professionals is already a commonly used approach in health chatbot development (i.e., Beaudry et al. 2019; Potts et al. 2021) and it is one way to address needs in content.

However, to propose realistic and useful solutions, healthcare professionals participating in design processes as part of multidisciplinary teams need to understand the possibilities and limitations of technologies being used, as well as requirements of a user‐engaging chatbot. Even though good information and guidelines on chatbot conversational design, in general, are available (Hall 2018; McTear 2021; Moore and Arar 2019), practical requirements targeted specifically to mental health chatbot design are often reported in research articles as a result of chatbot development projects and appear fragmented. In addition, design knowledge tends to be abstract in nature, and therefore not easy to implement directly (Ahmad et al. 2022). Previous literature reviews do not focus on conversational design of mental health chatbots (Curtis et al. 2021; Silva and Canedo 2022). For these reasons, there is a need to review what is known about mental health chatbot conversational design, and how this knowledge can serve chatbot development.

## 2 The Review

### 2.1 Aim

This review aims to identify and synthesise recommendations and guidelines for mental health chatbot conversational design. The following research questions are addressed: (1) What kind of guidelines and recommendations exist for mental health chatbot conversational design? And, (2) what kind of requirements do the current guidelines and recommendations set for mental health‐promoting chatbot development?

### 2.2 Design

Integrative review was chosen to conduct as it allows to include diverse methodologies, and data from theoretical and empirical literature (Toronto, Remington, and EBSCOhost 2020; Whittemore and Knafl 2005). This review synthesises literature from various sources, aiming to explore conversational design of mental health chatbots broadly and update knowledge in this rapidly changing field (Toronto, Remington, and EBSCOhost 2020). Whittemore and Knafl's (2005) framework, consisting of (1) problem identification, (2) literature search, (3) data evaluation, (4) data analysis and (5) presentation, was followed. This makes it possible to explore conversational design of mental health chatbots broadly, and still systematically, in line with the most recent knowledge. As applicable, this integrative review is reported according to the preferred reporting items for systematic review and meta‐analyses (PRISMA) (Page et al. 2021) (File S1; PRISMA 2020 checklist). The protocol of this review was not published.

### 2.3 Search Methods

To clearly define concepts, research questions and exclusion and inclusion criteria (Toronto, Remington, and EBSCOhost 2020), the search string was created by using PICOC method, which covers population, intervention, comparison, outcome and context (Silva and Canedo 2024; Wohlin et al. 2012). In this review, concepts were organised as follows: P = text‐based chatbots, I = conversational design, C = not applicable as there is no comparison between groups, O = guidelines or recommendations and C = mental health. Health and mental health are intertwined, so in order to gain comprehensive search results, the searches initially covered publications concerning both health and mental health chatbots.

Initial searches were run to see the relevance of keywords and to identify possible missing words. The university librarian was consulted in the preparation phase and before the final search was conducted (Toronto, Remington, and EBSCOhost 2020). Relevant databases including multidisciplinary databases, health sciences and computing were chosen. In integrative review, it is recommended to use more than one search strategy to obtain comprehensive search results (Whittemore and Knafl 2005). After systematic search of the databases, the second part of the search is recommended to cover relevant literature published outside of the peer‐reviewed journals as grey literature (Toronto, Remington, and EBSCOhost 2020). Additionally, as this review covers a multidisciplinary topic, it is important to note that software engineering insights and experiences are often published as grey literature (Garousi et al. 2020).

There are no established practices for grey literature search, but Google search engines, grey literature databases, websites and contacting subject experts can be used (Godin et al. 2015; Toronto, Remington, and EBSCOhost 2020). In the field of nursing science, previous integrative reviews have used Google Scholar in secondary searches to complement database searches (Duangjina et al. 2024) and find relevant grey literature (Appleby, Cowdell, and Booth 2021). Hence, the search strategy of this study had two steps: a comprehensive database search and secondary search from Google Scholar aiming to identify additional relevant publications.

The literature search was conducted on 20 February 2023 in PubMed, Scopus, ACM Digital Library and EBSCO databases including APA PsycINFO, CINAHL, APA PsycArticles and MEDLINE. To attain the most current data, the search was updated on 5 October 2023 with the same search strategy. The search was limited to last 10 years. The full search strategy can be found in File S2; Searches from databases.

A secondary search was carried out in Google Scholar on 30 May 2023 with the keywords: health, text based, chatbots, conversational design and requirements. The search was sorted by relevance; out of 4300 results, the first 100 were screened by the first author H.N. (Abd‐Alrazaq et al. 2020; Godin et al. 2015; Toronto, Remington, and EBSCOhost 2020). Based on this tentative screening, the second author A.‐K.V. read relevant articles and a decision about including them was discussed. A secondary search yielded two publications: one from the search result and another hand‐picked from its reference list.

### 2.4 Inclusion and Exclusion Criteria

The criteria for eligibility were (1) research articles, conference proceeding and expert opinions concerning text‐based chatbots, (2) publications that describes aspects of conversational design, (3)publications that provides recommendations, tips or guidelines that could be utilised in chatbot conversational design processes and (4) the area of use is health or mental health. Exclusion criteria were as follows: (1) purely usability or feasibility study, (2) technical description, (3) only conference abstract is available, (4) generative artificial intelligence was used to generate chatbot's responses, (5) article is about embodied conversational agents, (6) literature review or study protocol and (7) full text not available. In full‐text phase, articles concerning only health chatbots, but not mental health, were excluded.

### 2.5 Search Outcome

**FIGURE 1:**
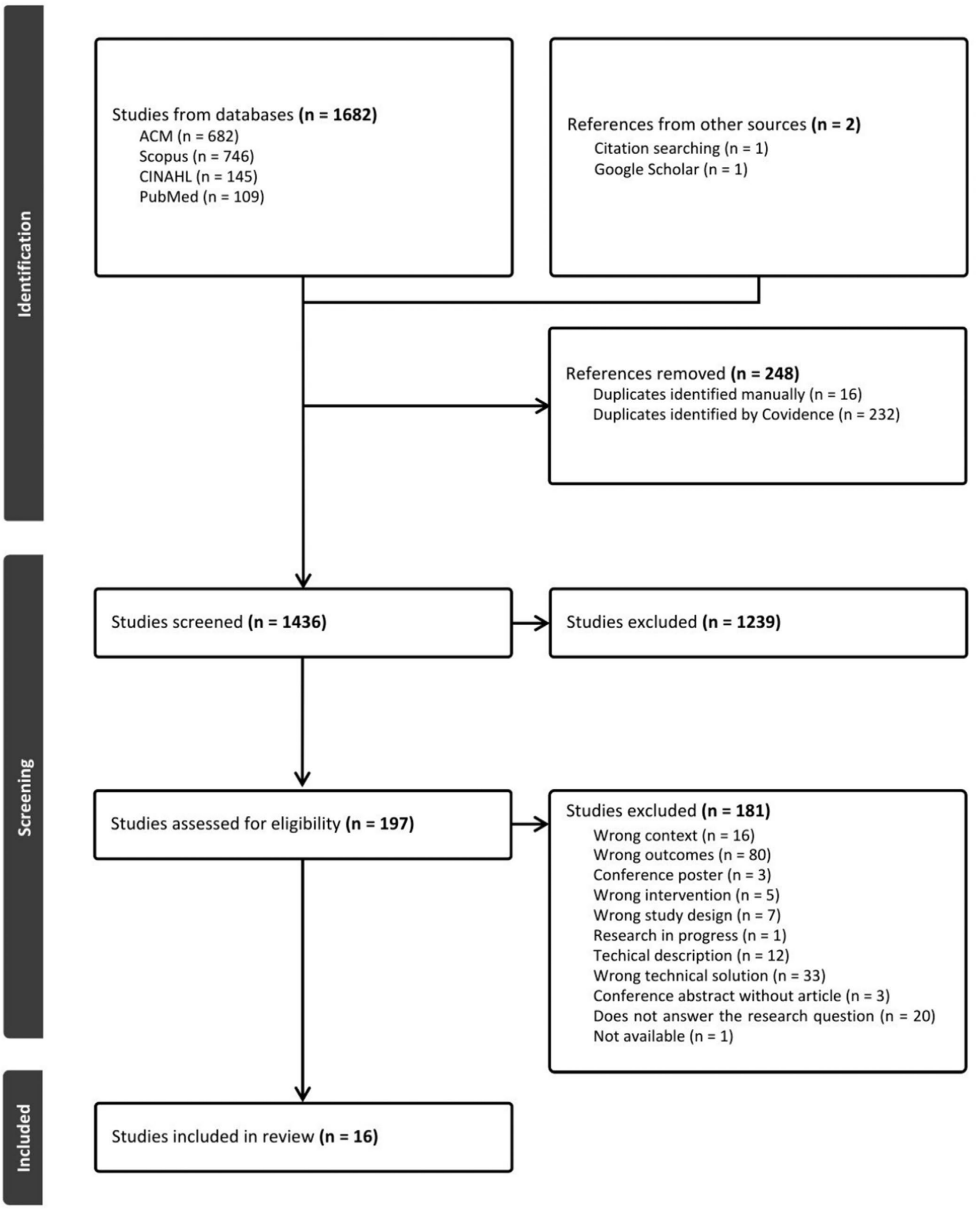
PRISMA flowchart describing the study identification and selection.

Search results (primary search *n* = 1311, secondary search *n* = 2 and updated search *n* = 371 records) were exported to Covidence software. After duplicates (*n* = 248 in total) were removed, two authors (H.N. and A.‐K.V.) independently screened titles, abstracts and full texts. Decisions about excluding and including articles were made based on predefined criteria. Conflicts at each stage were discussed, and in concluding phase, the last author's (L.K.) opinion was asked. The PRISMA flowchart of the process provided by Covidence software is shown in Figure 1 (Page et al. 2021). Cohen's kappa calculation by Covidence was used to assess interrater reliability. On the primary search, Cohen's kappa values were 0.44 (moderate) on the title and abstract screening and 0.57 (moderate) on the full‐text screening. The updated search values were 0.53 (moderate) and 1.0 (almost perfect), respectively (Landis and Koch 1977). Despite the predefined inclusion and exclusion criteria, broad and multidisciplinary research topics, diversity of screened articles and information provided in titles and abstracts may affect interrater reliability (Belur et al. 2021).

### 2.6 Quality Appraisal

The quality appraisal was conducted by two researchers (H.N. with A.‐K.V. or L.K.) individually. Joanna Briggs Institute Critical Appraisal Tools' Finnish versions by the Nursing Research Foundation (NRF) were used (Hotus. 2023). Publications were grouped based on suitable JBI critical appraisal tools. Three of them were evaluated as quasi‐experimental studies, three as expert opinion papers, 11 as qualitative research articles and one as a cross‐sectional study. Both qualitative and quasi‐experimental or cross‐sectional criteria were applied in two mixed‐methods studies to cover the whole research. Quality appraisal results by two evaluators were compared and any disagreements were discussed. None of the articles were excluded due to low quality (File S3; Quality appraisal).

### 2.7 Data Abstraction

Following data were extracted from each selected article: author, year and country, aims/objectives, methods, target group of chatbot, participants of the original study, domain area/content, main results or output and publication type (File S4; Included articles). Data were abstracted by H.N. alone.

### 2.8 Analysis

In integrative review, the data analysis stage aims to interpret primary sources thoroughly and without bias, and to synthesise the evidence in an innovative way (Whittemore and Knafl 2005). The analysis was done with Atlas.ti software version 23 for qualitative analysis.

Thematic analysis (TA) by Braun and Clarke (2022) was used to qualitatively analyse chosen articles. Opposite to the positivist approach often taken in literature reviews, the epistemological stance taken in this study was more interpretive (Harsh 2013). TA's flexibility enabled inductive analysis which captured semantic meanings of the recommendations at the beginning of the process and offered a possibility for interpretations of latent meanings in theme development in the latter stages of the analysis (Braun and Clarke 2022).

Analysis started with a thorough familiarisation of the selected articles. As this review aims to analyse actual recommendations presented in recent publications, each recommendation item was extracted and recommendations with similar semantic meaning were merged. After going through all recommendation items, they were coded inductively with the intention of capturing and evoking their meaning. Codes with shared meanings were developed further as subthemes. During the process, validity of coding and subthemes was ensured by going back to articles and checking original meaning and content of recommendation items. This also confirmed that each subtheme is supported by data from several sources. The analysis proceeded iteratively until three overarching themes were developed to represent the findings. This process was carried out by H.N. (Braun and Clarke 2022).

## 3 Results

### 3.1 Description of Selected Studies

Sixteen articles published in 2018–2023 were included in this review, including eight research articles, seven conference papers and one publication based on a development project (Table 1).

**TABLE 1:** Characteristics of the studies (*n* = 16) included in the integrative review.

Authors and country	Domain area	Target group of chatbots developed	Research design/article type	Publication type
Ahmad et al. (2022), Germany	Mental health	na	Design science research (DSR)	Research article
Cameron et al. (2018), UK	Mental health	Employees	Recommendations based on chatbot development process	Conference paper
De Souza et al. (2022), Brazil	Mental health, depression	na	Mixed‐methods study	Conference paper
Han et al. (2023), USA	Mental health, posttraumatic stress disorder	Individuals with PTSD	Qualitative study	Research article
Haque and Rubya (2023), USA	Mental health	na	Exploratory observation study	Research article
Kang et al. (2023), New Zealand	Mental health, to cope with stress and isolation due to COVID‐19 restrictions	Adolescents	Qualitative study	Research article
Kuhlmeier et al. (2022), Germany	Mental health, depression	Youth and young adults	Design science research (DSR)	Conference paper
Lee et al. (2019), the Netherlands	Mental health, self‐compassion	na	Mixed‐methods study	Conference paper
Lee et al. (2020), USA and Japan	Mental health	na	Mixed‐methods study	Conference paper
Lopatovska et al. (2022), USA	Mental health	Adolescents	Participatory design study	Conference paper
Maenhout et al. (2021), Belgium	Health promotion, mental well‐being	Adolescents	Mixed‐methods study	Research article
Mauriello et al. (2021), USA	Mental health, stress management	General public (healthy people dealing with daily stressors)	Mixed‐methods study	Research article
Peltola et al. (2023), Finland	Mental health, psychological flexibility and well‐being	Adolescents	Mixed‐method study	Conference paper
Potts et al. (2022), UK	Health and mental health	General public	Recommendations based on chatbot development process	Project report/book
Prakash et al. (2020), India	Mental health	na	Qualitative study	Research article
Ryu et al. (2020), South Korea	Mental health, anxiety and depression	Older adults	Mixed‐methods study	Research article

### 3.2 Findings

Based on this review, there are several recommendations for designing mental health chatbots. However, in addition to lists compiled, some research articles mention single‐design implications and recommendations separately.

#### 3.2.1 Types of Recommendations and Guidelines for Mental Health Chatbots

In the studies selected for this review, two types of recommendations or guidelines were identified. Firstly, there were those addressing the whole chatbot design and development process, covering, that is, chatbot personality, technical aspects, features and language used (Ahmad et al. 2022; Cameron et al. 2018; De Souza et al. 2022; Han et al. 2023; Lee et al. 2019; Maenhout et al. 2021; Mauriello et al. 2021; Potts et al. 2022; Prakash et al. 2020; Ryu et al. 2020). They provide a good overview of the many dimensions that need to be considered when designing and developing a mental health chatbot.

Recommendations in the second group were more focused, targeting self‐disclosure (Lee et al. 2020), conversational content (Kang et al. 2023; Lopatovska et al. 2022), balanced persuasion and trust (Haque and Rubya 2023) and interaction (Kang et al. 2023; Peltola et al. 2023), as well as personalisation and customisation (Haque and Rubya 2023; Kuhlmeier et al. 2022).

#### 3.2.2 TA of Recommendations and Guidelines

**FIGURE 2:**
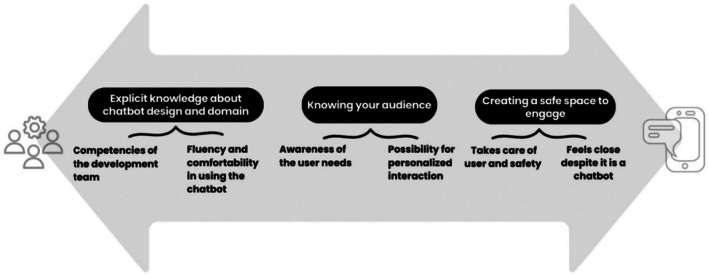
Main themes (above) and subthemes (below) developed in thematic analysis of recommendations and guidelines for mental health chatbot conversation design.

In following chapters, the themes developed during reflexive TA are presented. Overarching themes were ‘Explicit knowledge about chatbot design and domain’, ‘Knowing your audience’ and ‘Creating a safe space to engage’ (Figure 2, Table 2, File S5; Codes, subthemes and themes).

**TABLE 2:** Main themes and their characteristics.

Main theme	Characteristics
Explicit knowledge about chatbot design and domain	Identifies the need for mental health chatbot development teams to possess a wide range of specialised knowledge reached by multidisciplinary cooperation
Knowing your audience	Indicates the importance of knowing the user requirements beforehand but also gaining information during chatbot usage. Demand for personalisation and tailoring based on information gathered was recognised from the reviewed recommendations
Creating a safe space to engage	Collates both social and technical factors creating the experience of pleasantness and willingness to engage with chatbot use as well as safety issues

Underlining the multidimensionality of this topic and the process of chatbot development, themes were arranged as a continuum: technical, practical and development team‐oriented insights on the left hand, continuing to right where human factors of interaction are strongly present (Figure 2).

In chapters below, an overview of each main theme and subtheme is provided. Table 3 shows the codes associated with each subtheme.

**TABLE 3:** Codes associated with subthemes.

Subtheme	Codes forming this subtheme
1a. Competencies of the development team	Well‐organised authoring process Content considerations Chatbot personality and language
1b. Fluency and comfortability in using the chatbot	Improving interaction User experience of conversation Visuality
2a. Awareness of the user needs	Addressing the needs of the target group Chatbot gathering information
2b. Possibility for personalised interaction	Chatbot suitable for user's personality Demand for therapist‐like skills
3a. Takes care of user and safety	From digital encounter to human contact Informing users Proactive support Security
3b. Feels close despite it being a chatbot	Feeling of mutual closeness Inclusiveness Human‐like but not too much

**Main theme 1: Explicit knowledge about chatbot design and domain**

The first overarching theme identifies the need for mental health chatbot development teams to possess a wide range of specialised knowledge. It highly supports multidisciplinary teamwork. This main theme has two subthemes: competencies of the development team and fluency and comfortability in using the chatbot.

**Subtheme 1a: Competencies of the development team**

*Well‐organised authoring process*

Defining the explicit goal of the chatbot , modality, use of external knowledge (Potts et al. 2022), identifying stakeholders, the target audience and the limitations of the application (Potts et al. 2022; Prakash et al. 2020) are guiding the following phases of the process. It is advised to work together with the target group (Cameron et al. 2018), domain experts (Han et al. 2023; Potts et al. 2022) and potential users (Potts et al. 2022).

*Content considerations*

In mental health chatbot conversations, providing information about the topic is one of the key components (Ryu et al. 2020). Using and referring to reliable sources (Han et al. 2023; Maenhout et al. 2021) is important. A user should be able to ask questions (Maenhout et al. 2021) and report feelings and thoughts (Ryu et al. 2020) but also more fixed content is needed to meet the user's needs and execute the intervention intended (Han et al. 2023; Kuhlmeier et al. 2022). To address these requirements, and to maintain user engagement, variety is needed in content (Han et al. 2023). Interaction and reinforcement in educational conversations can be gained, for example, with quizzes related to the topic (Ryu et al. 2020). Some of the recommendations illustrate the interconnectedness of health and mental health. Mental health information and psychological strategies can be provided in a compassionate way (Lopatovska et al. 2022), and combined with other life advice (Kang et al. 2023). When mental health chatbot suggests physical activities, user's abilities to perform it need to be considered (Haque and Rubya 2023).

*Chatbot personality and language*

Coherent ways of interaction and chatbot personality are aspects which require planning upfront, considering the potential user group. Especially in articles covering research on chatbots targeted at young people, colloquial language was suggested, but it should not try to mimic the way adolescents speak (Maenhout et al. 2021; Peltola et al. 2023). According to Peltola et al. (2023), encouraging needs to be done carefully: They advise avoiding generic and commonly used phrases and excessive number of encouraging messages.

Too many and superfluous questions can be annoying and might seem inquisitive (Peltola et al. 2023). A recommended way of conversation is described as positive and nonjudgemental (Maenhout et al. 2021), including some conversational delights (Cameron et al. 2018) in line with the personality of the chatbot. Use of humour is suggested (Cameron et al. 2018; Maenhout et al. 2021; Prakash et al. 2020) as it may help build a connection (Cameron et al. 2018). Too complicated vocabulary and oppressive expressions should be avoided (De Souza et al. 2022).

**Subtheme 1b: Fluency and comfortability in using the chatbot**

*Improving interaction*

Interaction is recommended to be improved by several technical means, including recognising the time of day (Cameron et al. 2018; Ryu et al. 2020) and context (Cameron et al. 2018), and possibility of pausing and restarting the conversation when needed (De Souza et al. 2022). Long‐ and short‐term memory can prevent repetition and build a relationship (Cameron et al. 2018) but also help to ensure that responses fit precisely to the user's input (Han et al. 2023). To create the feeling of conversation, it is advisable to give the user proper time to read chatbot responses and use typing indicators (Cameron et al. 2018).

*User experience of conversation and visuality*

Conversation is often read from the smartphone screen, which demands short and concise pieces of text sent at once (Peltola et al. 2023). It is recommended to engage in small talk (Maenhout et al. 2021), which can also be used in gathering nonsensitive information (Lee et al. 2020). The possibility of writing free‐text responses is desirable (Kang et al. 2023; Lee et al. 2019; Maenhout et al. 2021; Prakash et al. 2020). However, button‐based solution was preferred by Ryu et al. (2020) in their study with older adults.

Eliminating errors is important (Kang et al. 2023), and chatbot should be capable of responding properly in case of unexpected user input or not understanding (Cameron et al. 2018; Potts et al. 2022), or even if the internet connection is lost (De Souza et al. 2022). Intuitive interface (Ryu et al. 2020) with appropriate amount of visual elements to bring variety is recommended (Cameron et al. 2018; De Souza et al. 2022; Haque and Rubya 2023; Maenhout et al. 2021; Peltola et al. 2023).

**Main theme 2: Knowing your audience**

The second main theme illustrates the importance of knowing the user requirements beforehand but also gaining information during chatbot usage. This main theme contains two subthemes: awareness of the user needs and possibility for personalised interaction.

Features of both personalisation and tailoring were recognised from the reviewed recommendations. Personalisation concerns providing specific content or services for a single user. Tailoring in turn means providing content designed for a specific user group (Oinas‐Kukkonen and Harjumaa 2009).

**Subtheme 2a: Awareness of the user needs**

*Addressing the needs of the target group*

Identifying the stakeholders and user group is followed by getting to know the users' preferences and needs (Lee et al. 2019; Potts et al. 2022) with coproduction approach (Potts et al. 2022). Understanding the problems in which users want chatbot to support them (Lopatovska et al. 2022) helps to create relatable conversations (Kang et al. 2023) and choose content that is perceived as useful for users (Maenhout et al. 2021).

*Chatbot gathering information*

In addition to gathering knowledge on users at the development phase, it is suggested to be done also during the chatbot usage. Condition, mood or symptoms can be assessed with scales and questions, and it is particularly important with chronic conditions and vulnerable groups. It also helps the users to monitor their own well‐being (Han et al. 2023). Identifying keywords from the user input can help the chatbot address the user's situation better with follow‐up questions (Ryu et al. 2020). Asking for feedback about users' condition before and after a conversation helps to check the suitability of the intervention (De Souza et al. 2022). Feedback about the interaction enables improving the chatbot (De Souza et al. 2022; Potts et al. 2022) and preventing disappointments (Prakash et al. 2020).

**Subtheme 2b: Possibility for personalised interaction**

*Chatbot suitable for user's personality*

The need for personalisation of conversations in different ways has been identified especially in the most recent articles: In these data, all from years 2022 to 2023 (Ahmad et al. 2022; De Souza et al. 2022; Haque and Rubya 2023; Kuhlmeier et al. 2022). It was suggested that user could choose, for example, chatbot's level of anthropomorphism, social role (Ahmad et al. 2022) and avatar, name, gender (De Souza et al. 2022; Kuhlmeier et al. 2022) and way of speaking (De Souza et al. 2022; Kuhlmeier et al. 2022). Moreover, Kuhlmeier et al. (2022) discuss the importance of personalising chatbot content to meet user needs and achieve relevant conversations.

*Demand for therapist‐like skills*

The increasing knowledge of chatbots and the advances in technology are bringing high expectations for mental health chatbots. To address those, learning algorithms and scoring can be used to select interventions and identify severe situations, in which chatbot is not the right aid (Mauriello et al. 2021). An important skill is the ability to suggest interventions (De Souza et al. 2022; Mauriello et al. 2021). Before that, it is needed to identify the reasons for possible symptoms and previous attempts to solve the situation (De Souza et al. 2022). Conversations are expected to be high in quality: user's condition needs to be taken into account when designing persuasive techniques (Haque and Rubya 2023), and offer guided interventions as well as possibility to just vent and reflect thoughts by writing (Kang et al. 2023; Mauriello et al. 2021). Users should feel heard and understood (Ahmad et al. 2022; Kang et al. 2023), while the chatbot keeps the conversation going without feeling rushed or under pressure (De Souza et al. 2022).

**Main Theme 3: creating a safe space to engage**

The third overarching theme considers factors creating the experience of pleasantness, safety and willingness to engage with chatbot use. From the user's point of view, safety is a multidimensional concept, including experience as well as technical solutions.

**Subtheme 3a: Takes care of user and safety**

*From digital encounter to human contact*

To balance technology usage, ways to guide the users from digital encounters to human contact were identified. A trusted person whom to contact in an emergency situation could be named (De Souza et al. 2022; Prakash et al. 2020). Chatbot should support the discovery of human contacts (Prakash et al. 2020) and care (De Souza et al. 2022) and make helpline numbers easily available (Potts et al. 2022; Prakash et al. 2020). In addition, the prevention of unhealthy usage of technology was brought up, so it is needed to consider when and how to limit chatbot usage (Haque and Rubya 2023; Prakash et al. 2020).

*Informing users, proactive support and security*

A chatbot must be transparent about its capabilities and limitations (Han et al. 2023; Potts et al. 2022), and provide information about the coaching approach and the experts who created the chatbot (Haque and Rubya 2023). In addition, a clear description of the usage of any collected data (De Souza et al. 2022; Prakash et al. 2020) is recommended to offer. Four articles suggested that chatbots do proactively contact users, for example, with recommendations and reminders (Ahmad et al. 2022; De Souza et al. 2022; Maenhout et al. 2021; Ryu et al. 2020). Data security issues, including the possibility of logging in with a password (De Souza et al. 2022), having discussions anonymously (Maenhout et al. 2021) and applying other safety measures to protect user data (De Souza et al. 2022), were brought up in the articles. Importance of transparency in informing about privacy and safety policies was also mentioned (Ahmad et al. 2022; Haque and Rubya 2023).

**Subtheme 3b: Feels close despite it being a chatbot**

*Feeling of mutual closeness and inclusiveness*

Feeling of mutual closeness can be promoted with greetings, using the person's name (Cameron et al. 2018; De Souza et al. 2022; Maenhout et al. 2021) and asking questions to get to know each other (Maenhout et al. 2021; Peltola et al. 2023). Shared storytelling can be executed even without complex AI (Lee et al. 2019). Expressing empathy is suggested for chatbots (De Souza et al. 2022; Kang et al. ; Maenhout et al. 2021; Prakash et al. 2020). Inclusiveness related to age (Lee et al. 2019), gender (Lee et al. 2019; Peltola et al. 2023), culture (Cameron et al. 2018; Lee et al. 2019; Potts et al. 2022), location, income and literacy level needs to be considered (De Souza et al. 2022).

*Human‐like but not too Much*

Conversations can be valuable even if there is artificiality in it (Peltola et al. 2023) when it is adaptable and thorough (Prakash et al. 2020). Conversation designers need to consider how the chatbot expresses its human‐like features, for example, feelings (Lee et al. 2019) or ‘personal experiences’ (Peltola et al. 2023), in a way it does not cause negative emotions, such as eeriness (Prakash et al. 2020).

##### 3.2.2.1 Practical Insights on Mental Health Chatbot Conversational Design

Based on TA described in previous chapters, the aspects requiring consideration during the mental health chatbot conversational design process are outlined. The main practical insights gained from this review are summarised as a checklist for mental health chatbot developers in Table 4.

**TABLE 4:** The main practical insights gained from this review for designing mental health chatbot conversations.

In mental health chatbot conversational design …	
plan the process carefully beforehand	target group's needs and preferences theoretical background and references team involving experts from suitable fields chatbot personality and language
make sure the conversation is fluent and pleasant	chatbot's relatability and usefulness for the target audience abilities and limitations of the chatbot personalisation visuality and variety in the conversation chatbot's capabilities to handle errors suitable amount of human likeness and empathy
foster safety	technical safety measures availability of human support when needed suitable level of chatbot's proactivity in interaction informing users about the chatbot limitations, data security and the course of conversation

## 4 Discussion

This review analysed design guidelines for mental health chatbots. Firstly, we wanted to investigate what kind of guidelines and recommendations exist in mental health chatbot conversational design. The second research question was posed to interpret what kind of requirements these recommendations set for mental health chatbot development. Drawing on TA, as well as data extracted from the selected studies, we were able to answer our research questions.

Knowledge on this topic is very recent, which is reflected in the publication years of the data in this review: only two of the articles were published before 2020. Recommendations evolve as a result of chatbot development and research. For this reason, they appear fragmented. Using reflexive TA (Braun and Clarke 2022), three overarching themes were developed: creating a safe space to engage, knowing your audience and explicit knowledge about chatbot design and domain. They describe important aspects to pay attention to in mental health chatbot conversational design.

Support provided by a chatbot or a mental healthcare professional shall not be equated and implementing mental health chatbots needs to be carried out with caution. However, interesting similarities between recommendations reported in reviewed articles and previous knowledge about components of high‐quality mental health nursing can be found: respect and interest towards the uniqueness of user's personality and situation (Dziopa and Ahern 2009; Gunasekara et al. 2014), listening skills, identifying and responding to user's individual needs, providing support, addressing safety concerns (Bucci, Schwannauer, and Berry 2019) and being available (Dziopa and Ahern 2009) were present also in our results. This brings novel insights into the development of mental health chatbot conversations, as well as further justifies the importance of mental health professionals' involvement in design processes (Ronquillo et al. 2021).

Expectations towards mental health chatbots’ conversational capabilities are high: they should be empathic and respond to a user in a variety of ways (Abd‐Alrazaq et al. 2021). Respectively, other health chatbots may serve more practical purposes, such as sharing information, providing behavioural prompts (Ashton et al. 2023) or collecting information with a health questionnaire (Tominaga et al. 2021). These types of applications do not necessarily require advanced conversation skills and a feeling of natural conversation (Höhn and Bongard‐Blanchy 2020) as chatbots targeted to mental health.

As a result of their systematic literature review, Silva and Canedo (2022) presented a conceptual map of chatbot conversations. Unlike ours, their review did not focus only on health chatbots, and the review method was different. However, similarities exist in results. For example, when chatbot's purpose is to promote well‐being, it is suggested to use empathetic messages including some humour to support the experience of intimacy and encouragement. Graphical media were also mentioned as a way to express emotionality and support (Silva and Canedo 2022). In our review, visuality was one of the codes appearing in the data.

The topic of personalising chatbots and other digital health interventions is worth paying attention to since it appears to be a current topic (Hornstein et al. 2023). As mentioned in the results section, recommendations concerning personalisation were extracted from very recent articles. In their systematic review published in 2023, Hornstein et al. proposed a conceptual framework of personalisation in digital mental health interventions describing dimensions of personalisation, namely, personalisation of content, its order, guidance and communication. Two of those dimensions reported in Hornstein et al.'s article were also presented in the results of this review. Dimension of communication appears as reminders to engage with the chatbot or perform a certain behaviour (in this review labelled with the code ‘Proactive support’, e.g., Ahmad et al. 2022 and Ryu et al. 2020). Dimension of guidance is reflected in recommendations to provide support for finding human care (e.g., De Souza et al. 2022; Haque and Rubya 2023) or relevant helpline numbers being available (Potts et al. 2022; Prakash et al. 2020), which are here interpreted to be under code ‘From digital encounter to human contact’. This review focuses on conversational design, which adds an additional layer of personalisation: the chatbot's suitability for users' personalities and the possibility of choosing or adapting it to meet communication goals.

To be broadly used or recommended by healthcare professionals, novel solutions like mental health chatbots need to be high in quality, effective, safe and affordable (World Health Organization 2024). Health Technology Assessment (HTA) by WHO is a tool used by several countries to assess new technologies and support decision‐making. It outlines aspects which are useful to consider also during the chatbot development process. Several aspects covered in WHO's 2015 Global Survey on HTA by National Authorities were reflected also in the results of this review: safety, equity issues, ethical issues, acceptability to healthcare providers and acceptability to patients (World Health Organization 2015).

### 4.1 Implications to Practice

This review combines recommendations from recent publications that are helpful for mental health professionals participating in chatbot development processes, and also for those teams in which healthcare professionals are not closely involved. When applying these results, it is worth noticing that the articles included in this review are mostly from high‐income countries (*n* = 14) (Metreau, Young, and Shwetha 2024).

### 4.2 Limitations and Strengths

It is possible that despite careful planning of the literature search and support of university librarians, some relevant articles may not be included in this review. Only those articles published in English were searched. In secondary search from Google Scholar, only one author conducted the initial screening and only references freely available were considered.

There may have been some aspects of relevance missed because articles regarding generative AI or large language models were excluded. However, limiting this review to rule‐based chatbots offered a possibility to explore this topic in depth.

Literature search was carried out in multidisciplinary databases and databases covering health sciences and computing. The study design, study setting or country was not restricted, and conference papers were also considered. For these reasons, review can be considered comprehensive in relation to topic and aim of this review. Two reviewers independently screened the studies and assessed their quality. Selection was based on consensus.

In reflexive TA, interpretation is embedded in analysis process (Braun and Clarke 2022). Researchers were aware of inevitable subjectivity when adopting interpretive approach (Harsh 2013). The analysis is conducted by the first author (H.N.), who interpreted the data based on her previous knowledge and experience on mental health chatbot content and conversation development, healthcare practice and nursing science. However, a multidisciplinary group, including experts in mental healthcare, computing, human–computer interaction and health application development, contributed to other phases of this review.

## 5 Conclusion

A mental health chatbot conversation consists of several phases, from informing the user to encouraging them to disclose or discuss sensitive matters. These phases require different emphases in conversational design. This review brings insights into creating conversations for mental health chatbots and points out special characteristics of mental health chatbot recommendations.

In this review, both general and more specific recommendations for mental health chatbot development were identified. As a result of a TA, three main themes were developed to represent requirements set forth by current guidelines and recommendations for the development of mental health chatbots. Main theme ‘Explicit knowledge about chatbot design and domain’ covers the profound prework to be done in advance to network with the right professionals and possible end users and consider many solutions guiding the conversational design, as well as consider the technical means to ensure fluent user experience. Main theme ‘Knowing your audience’ highlights the importance of awareness of user needs and preferences and demand on personalised interaction. The third main theme, ‘Creating a safe space to engage’, shows that in chatbot context, safety does not only mean data security with technical means but also perception of warm and supporting interaction.

In addition, there are features that need to be especially considered in chatbots for mental health promotion and care. Human support is recommended to be available by providing information about services near the user, relevant helpline numbers or the possibility of contacting trusted person quickly in case of emergency. Suitable amount of proactivity and visual elements are important. The needs of the target group need to be considered carefully to be able to develop a chatbot with required abilities to precisely address them and provide useful and pleasant human–chatbot encounters.

## Author Contributions

Heidi Nieminen, Anna‐Kaisa Vartiainen, Raymond Bond, Emilia Laukkanen, Maurice Mulvenna and Lauri Kuosmanen have made substantial contributions to conception and design, acquisition of data or analysis and interpretation of data and involved in drafting the manuscript or revising it critically for important intellectual content and given final approval of the version to be published. Each author should have participated sufficiently in the work to take public responsibility for appropriate portions of the content and agreed to be accountable for all aspects of the work in ensuring that questions related to the accuracy or integrity of any part of the work are appropriately investigated and resolved.

## Conflicts of Interest

The authors declare no conflicts of interest.

## Peer Review

The peer review history for this article is available at https://www.webofscience.com/api/gateway/wos/peer‐review/10.1111/jan.16762.

## Supporting information


File S1.

File S2.

File S3.

File S4.

File S5.

## Data Availability

Data sharing is not applicable to this article as no new data were created or analyzed in this study.
