# Supplementary material for: Recommendations for Mental Health Chatbot Conversations: An Integrative Review
Source: J Adv Nurs. 2025 Jan 22;81(10):6169–82. doi: 10.1111/jan.16762 (PMC12460983; doi:10.1111/jan.16762)
Supplement: Supplementary file 2 — File S2. [file JAN-81-6169-s004.docx]

**Supplementary file 2. Searches from databases**

Database searches were conducted on 20 February 2023 and updated on 5 October 2023. Updating the search covered the period between the first and second searches.

**Scopus: 554, rerun 192**

( TITLE-ABS-KEY ( chatbot OR chatterbot OR "conversational agent*" OR "conversational interface" OR "conversational system" OR "dialogue system" ) AND TITLE-ABS-KEY ( health OR wellbeing OR "well being" OR well-being OR "mental health" ) AND ALL ( guidelines OR checklist OR "best practise*" OR "best practice*" OR principle* OR recommendat* OR heuristic* ) AND ALL ( "conversation*" OR "interaction" OR "dialogue" OR "conversational design" OR "conversation design" OR content ) ) AND ( EXCLUDE ( DOCTYPE , "re" ) ) AND ( LIMIT-TO ( PUBYEAR , 2023 ) OR LIMIT-TO ( PUBYEAR , 2022 ) OR LIMIT-TO ( PUBYEAR , 2021 ) OR LIMIT-TO ( PUBYEAR , 2020 ) OR LIMIT-TO ( PUBYEAR , 2019 ) OR LIMIT-TO ( PUBYEAR , 2018 ) OR LIMIT-TO ( PUBYEAR , 2017 ) OR LIMIT-TO ( PUBYEAR , 2016 ) OR LIMIT-TO ( PUBYEAR , 2015 ) OR LIMIT-TO ( PUBYEAR , 2014 ) ) AND ( LIMIT-TO ( LANGUAGE , "English" ) )

**EBSCO databases (CINAHL, APA PsycInfo, APA PsycArticles, MEDLINE): 134 (100 references exported, 34 duplicates automatically removed by database), rerun 45**

( chatbot OR chatterbot OR "conversational agent*" OR "conversational interface" OR "conversational system" OR "dialogue system" ) AND ( conversation* OR interaction OR dialogue OR "conversational design" OR "conversation design" OR content ) AND ( guidelines OR checklist OR "best practise*" OR "best practice*" OR principle* OR recommendat* OR heuristic* ) AND ( health OR wellbeing OR "well being" OR well-being OR "mental health" )

Limiters - Published Date: 20140101-20231231 Expanders - Apply equivalent subjects

Search modes - Boolean/Phrase

**PubMed: 83, rerun 26**

(((chatbot OR chatterbot OR "conversational agent*" OR "conversational interface" OR "conversational system" OR "dialogue system") AND (conversation* OR interaction OR dialogue OR "conversational design" OR "conversation design" OR content)) AND (guidelines OR checklist OR "best practise*" OR "best practice*" OR principle* OR recommendat* OR heuristic*)) AND (health OR wellbeing OR "well being" OR well-being OR "mental health")

**ACM: 574, rerun 108**

[[Abstract: chatbot] OR [Abstract: chatterbot] OR [Abstract: "conversational agent"] OR [Abstract: "conversational agents"] OR [Abstract: "conversational interface"] OR [Abstract: "conversational system"] OR [Abstract: "dialogue system"]] AND [[Abstract: conversation*] OR [Abstract: interaction] OR [Abstract: dialogue] OR [Abstract: "conversational design"] OR [Abstract: "conversation design"] OR [Abstract: content]] AND [[All: guidelines] OR [All: checklist] OR [All: "best practise"] OR [All: "best practises"] OR [All: "best practice"] OR [All: "best practices"] OR [All: principle*] OR [All: recommendat*] OR [All: heuristic*]] AND [[All: health] OR [All: wellbeing] OR [All: "well being"] OR [All: well-being] OR [All: "mental health"]] AND [E-Publication Date: (01/01/2014 TO 02/28/2023)]
