# Supplementary material for: Recommendations for Mental Health Chatbot Conversations: An Integrative Review
Source: J Adv Nurs. 2025 Jan 22;81(10):6169–82. doi: 10.1111/jan.16762 (PMC12460983; doi:10.1111/jan.16762)
Supplement: Supplementary file 5 — File S5. [file JAN-81-6169-s003.pdf]

Supplemetary file 5. Codes, subthemes and main themes

Main themes	Amount of subthemes under this main theme	Subthemes forming this main theme
**MAIN1** EXPLICIT KNOWLEDGE ABOUT CHATBOT DESIGN AND DOMAIN	2	**SUB1a** Competencies of the development team<br>**SUB1b** Fluency and comfortability in using the chatbot
**MAIN2** KNOWING YOUR AUDIENCE	2	**SUB2a** Awareness of the user needs<br>**SUB2b** Possibility for personalized interaction
**MAIN3** CREATING A SAFE SPACE TO ENGAGE	2	**SUB3a** Takes care of user and safety<br>**SUB3b** Feels close despite it is a chatbot
**Subthemes**	**Amount of codes supporting this subtheme**	**Codes forming this subtheme**
**SUB1a** Competencies of the development team	3	Well-organized authoring process<br>Content considerations<br>Chatbot personality and language
**SUB1b** Fluency and comfortability in using the chatbot	3	Improving interaction<br>User experience of conversation<br>Visuality
**SUB2a** Awareness of the user needs	2	Addressing the needs of the target group<br>Chatbot gathering information
**SUB2b** Possibility for personalized interaction	2	Chatbot suitable for user's personality<br>Demand on therapist-like skills
**SUB3a** Takes care of user and safety	4	From digital encounter to human contact<br>Informing users<br>Proactive support<br>Security
**SUB3b** Feels close despite it is a chatbot	3	Feeling of mutual closeness<br>Inclusiveness<br>Humanlike but not too much

Code	Amount of recommendations coded with this code (grounded)	Recommendation=quotation from original data	Reference
○ Addressing the needs of the target group	4		
	1	○ Adopt a co-production approach to generate solutions which address user needs	Potts et al. 2022
	1	○ Explore conversational solutions around teens’ everyday stressors	Lopatovska et al. 2022
	1	○ Avoidance of redundant answers	Maenhout et al. 2021
	1	○ Make the chatbot relatable to its target audience	Kang et al. 2023
○ Chatbot gathering information	7		
	1	○ Addressing anxiety and low mood with follow up questions and/or scales	Ryu et al. 2020
	1	○ Assessment and stability monitoring	Han et al. 2023
	1	○ Collect user feedback and harmonize service to prevent disappointments	Prakash & Das 2020
	1	○ Explicit feedback about interaction is asked	De Souza et al. 2022
	1	○ Explicit feedback about users mh is asked	De Souza et al. 2022
	1	○ Gathering relevant information to optimize interaction across users	Mauriello et al. 2021
	1	○ AI-based technigues or standard screening questionnaires to identify and screen complex conditions	Prakash & Das 2020
○ Chatbot personality and language	13		
	1	○ Avoiding cliché-like expressions especially when providing encouragement	Peltola ym. 2023
	1	○ Avoiding overt inquisitiveness	Peltola ym. 2023
	1	○ Conversation delights included	Cameron et al. 2018
	1	○ Fine-tuning the amount of encouragement in the messages	Peltola ym. 2023
	1	○ Lightness in dialogue	De Souza et al. 2022
	1	○ Non-judgmental	Maenhout et al. 2021
	1	○ Personality of CA is a key feature	Prakash & Das 2020
	1	○ Positive tone	Maenhout et al. 2021
	3	○ Use of humour and building a rapport	Cameron et al. 2018, Maenhout et al. 2021, Prakash & Das 2020
	2	○ Using colloquial language but not trying to imitate young peoples way of speaking	Maenhout et al. 2021, Peltola et al. 2023

○ Chatbot suitable for user personality	7		
	1	○ Chatbot's social role can be chosen	Ahmad et al. 2022
	1	○ Different language cues to different personalities	Ahmad et al. 2022
	1	○ Level of anthropomorphism can be adjusted by choosing the way of interaction	Ahmad et al. 2022
	1	○ Offer options to personalize the chatbot	De Souza et al. 2022
	1	○ Personalization based on user type	Haque & Rubya 2023
	1	○ Personalized chatbot character for relationship building	Kuhlmeier et al. 2022
	1	○ Personalized content to match user needs and preferences	Kuhlmeier et al. 2022
○ Content considerations	12		
	1	○ Adolescents are able to ask questions related to topics that are difficult to talk about with their parents	Maenhout et al. 2021
	1	○ Appropriate referrals	Maenhout et al. 2021
	1	○ User can report of thoughts, feelings, or behaviors	Ryu et al. 2020
	1	○ Combination of fixed and personalized content to maintain the structure	Kuhlmeier et al. 2022
	1	○ Emotional and cognitive support combined	Lopatovska et al. 2022
	1	○ Gamification and intrinsic motivation to engage	Ryu et al. 2020
	1	○ Follow established therapeutic resources and guidelines to generate content	Han et al. 2023
	1	○ Mixing psychological strategies with other life advice	Kang et al 2023
	1	○ Physical abilities noticed in exercise options	Haque & Rubya 2023
	1	○ Provide mental health information	Ryu et al. 2020
	1	○ Dynamic and tailored content	Han et al. 2023
	1	○ Trustworthy	Maenhout et al. 2021
○ Demand for therapist-like skills	11		
	1	○ Accurate answers	Maenhout et al. 2021
	2	○ Chatbot able to suggest interventions, but identify when more support is needed	Mauriello et al. 2021, De Souza et al. 2022
	1	○ Chatbot does not add pressure on the user	De Souza et al. 2022
	1	○ Check which protective factors have already been tried	De Souza et al. 2022
	1	○ Consider a multitude of coping and conversational styles	Mauriello et al. 2021
	1	○ Consider the range and severity of illnesses among the [illegible]	Haque & Rubya 2023
	1	○ Domain specific knowledge and therapeutic techniques	Ahmad et al. 2022
	1	○ Emphasize the nonhuman ability of chatbots to “listen” without interrupting	Kang et al 2023

		1	○ Go beyond the symptoms. Identify the determinants of suffering	De Souza et al. 2022
		1	○ Provides interaction options for uncertain subjective feelings	De Souza et al. 2022
○ Feeling of mutual closeness		12		
		1	○ ask questions and elicit responses to “talk” through a problem	Kang et al 2023
		3	○ CA uses the users name	Cameron et al. 2018, De Souza et al. 2022, Maenhout et al. 2021
		4	○ Express empathy	De Souza et al. 2022, Maenhout et al. 2021, Kang et al 2023, Prakash & Das 2020
		1	○ Flexible narratives for co-storytelling	Lee et al. 2019
		1	○ Introduction	Maenhout et al. 2021
		1	○ Self-disclosure if chatbot asks sensitive questions	Lee et al. 2020
		1	○ Showing interest by asking questions	Peltola ym. 2023
○ From digital encounter to human contact		10		
		2	○ Consider when and how to limit user interaction with chatbots	Haque & Rubya 2023, Prakash & Das 2020
		2	○ Encourage also human interaction	Haque & Rubya 2023, Prakash & Das 2020
		1	○ Provide shortcuts to reach out to contact persons quickly	De Souza et al. 2022
		1	○ Support the user to find human care	De Souza et al. 2022
		2	○ The helpline numbers relevant to the patient’s location should be made	Potts et al. 2022, Prakash & Das 2020
		2	○ User can name a contact persons for the event of crisis	De Souza et al. 2022, Prakash & Das 2020
○ Humanlike but not too much		5		
		1	○ Artificiality can be considered appropriate	Peltola ym. 2023
		1	○ Consider whether the conversational agent should have "personal experiences"	Peltola ym. 2023
		1	○ Emotional expressions are designed with caution as a continuum	Lee et al. 2019
		1	○ Enhancing humanness beyond optimal levels may trigger negative emotions	Prakash & Das 2020
		1	○ Intelligence of CA is a key feature	Prakash & Das 2020

○ Improving interaction	7		
	1	○ A correct time reference	Ryu et al. 2020, Cameron et al. 2018
	1	○ Analyse the complexity of language using text analysis tools	Cameron et al. 2018
	1	○ Appropriate delays and typing feedback/indicators need to be in place	Cameron et al. 2018
	1	○ Context awareness	Cameron et al. 2018
	1	○ Long term and short-term memory to build an intelligent relationship	Cameron et al. 2018
	1	○ Options to pause and restart the conversation	De Souza et al. 2022
	1	○ Consider that responses from a CA are contingent upon the user's input	Han et al. 2023
○ Inclusiveness	8		
	1	○ Accessibility (impairments, litteracy, income, location)	De Souza et al. 2022
	1	○ Tailor for different age groups	Lee et al. 2019
	2	○ Mindful of culture	Cameron et al. 2018, Lee et al. 2019
	1	○ Consider occupation	Lee et al. 2019
	2	○ Tailor for different genders, gender differences	Lee et al. 2019, Peltola ym. 2023
	1	○ Translations and cultural adaptations done well	Potts et al. 2022
○ Informing users	10		
	1	○ Ask for assistance with the app	Maenhout et al. 2021
	2	○ User is informed about any data collection, storage and use	De Souza et al. 2022, Prakash & Das 2020
	2	○ User is informed of technology limitations and abilities	Potts et al. 2022, Han et al. 2023
	1	○ Developers should communicate scientific evidence about the clinical effectiveness of their apps	Prakash & Das 2020
	1	○ Inform the estimated time of the dialogue	De Souza et al. 2022
	1	○ Provide information on the history of the developing organization/experts	Haque & Rubya 2023
	2	○ Transparent communication about safety and privacy to build trust	Ahmad et al. 2022, Haque & Rubya 2023
○ Proactive support	5		
	1	○ Chatbot proactively checks in on the user	Ahmad et al. 2022
	1	○ Non-embarrassing, non-identifiable alert messages	De Souza et al. 2022
	1	○ Notifications	Maenhout et al. 2021
	1	○ Reminders to perform a behavior or to use the system	Ryu et al. 2020
	1	○ Recommending activities	Ryu et al. 2020

○ Security	4		
	1	○ Address privacy and trust concerns	Han et al. 2023
	1	○ Anonymity	Maenhout et al. 2021
	1	○ Login with password	De Souza et al. 2022
	1	○ Security technologies applied	De Souza et al. 2022
○ User experience of conversation	17		
	1	○ Acknowledge what the user is saying	Kang et al 2023
	1	○ Developers should work on limiting the use of 'button press	Prakash & Das 2020
	1	○ Carefully planned design desicions (tone, person,	Potts et al. 2022
	1	○ Eliminate glitches	Kang et al 2023
	1	○ Free dialogue	Maenhout et al. 2021
	1	○ Intuitive and straightforward interface and interactions	Ryu et al. 2020
	1	○ Give users more opportunities to respond through free text	Kang et al 2023
	1	○ Provide users with more fixed responses and more opportunities for open input (if NLP is not an option)	Lee et al. 2019
	1	○ Use NLP, which will rely less on a pre-planned narrative arc and build more on what users say	Lee et al. 2019
	1	○ Concise messages	Peltola ym. 2023
	2	○ Plan chatbot response in case of unexpected inputs or chatbot not understanding	Cameron et al. 2018, Potts et al. 2022
	2	○ Short and precise interaction	Maenhout et al. 2021, Kang et al 2023
	2	○ Small talk for a reason	Lee et al. 2020, Maenhout et al. 2021
	1	○ Conversation ends nicely also without internet connection	De Souza et al. 2022
○ Visuality	7		
	3	○ Appropriate use of emojis	De Souza et al. 2022, Haque & Rubya 2023, Peltola ym. 2023
	1	○ Emoticons	Maenhout et al. 2021
	1	○ Use of icons to facilitate conversation	De Souza et al. 2022
	2	○ Variance in content and conversation	Cameron et al. 2018, Maenhout et al. 2021
○ Well organized authoring process	9		
	1	○ Carefully planned design desicions (tone, person, interaction, ext knowledge)	Potts et al. 2022
	1	○ Chatbot content and conversations are internally assessed before sign off	Potts et al. 2022

		1	○ Data provided by users is used wisely	Potts et al. 2022
		1	○ Explicitly specify the target audience and the limitations of the app	Prakash & Das 2020
		1	○ Focus on lowering barriers to authorship	Mauriello et al. 2021
		1	○ Identify stakeholders	Potts et al. 2022
		2	○ Involve domain experts (multidisciplinary team)	Potts et al. 2022, Han et al. 2023
		1	○ Observing the real conversations within a target group	Cameron et al. 2018
